# NADPH oxidase NOX4 is a glycolytic regulator through mROS-HIF1α axis in thyroid carcinomas

**DOI:** 10.1038/s41598-018-34154-8

**Published:** 2018-10-26

**Authors:** Ping Tang, Hao Dang, Jie Huang, Tao Xu, Ping Yuan, Jun Hu, Jian-feng Sheng

**Affiliations:** 1 Otorhinolaryngology Head and Neck Surgery, The Third Hospital of Mianyang(Sichuan mental health center), No. 190 The East Jiannan Road, Mianyang, 621000 Sichuan People’s Republic of China; 2 Department of Clinical Laboratory, The Third Hospital of Mianyang(Sichuan mental health center), No. 190 The East Jiannan Road, Mianyang, 621000 Sichuan People’s Republic of China; 3 Yunnan Jiehui Biotech Ltd., the KunMing economic and Technological Development Zone, No. 9 The Daxi Road, 650215, KunMing, Yunnan People’s Republic of China

## Abstract

The function of the NAD(P)H oxidases (NOXs) family member NOX4 is to generate reactive oxygen species (ROS), however, the molecular function of NOX4 has not been fully studied and waiting to be clarified. To elucidate the function of endogenous Nox4 in human thyroid carcinomas, papillomatosis thyroid cancer cells were used to study the cell growth by knocking down the expression of NOX4 and knocking out its functional partner p22phox/CYBA. As a result, the increasement of mitochondrial ROS(mROS) was abolished due to both knockdown of NOX4 and p22phox knockout in hypoxia, which destabilized HIF1α decreasing glycolysis and retarded cell growth. These data suggests that Nox4 is potent oncotarget due to its role in regulating glycolysis through mROS-HIF1α pathway, thereby mediating proliferation in thyroid carcinomas.

## Introduction

Papillary thyroid cancer (PTC) is the most common histologic type of human thyroid carcinoma that continues to be the most rapidly increasing cancer^1^. Although partially due to overdiagnosis because of increased use of advanced imaging techniques, occasionally they dedifferentiate into more aggressive and lethal thyroid cancers^2^. Therefore, investigating the underlying molecular mechanisms of PTC can provide promising biomarkers and therapeutic targets for early diagnosis and treatment, thus improving prognosis and survival quality of patients, especially those with aggressive tumor behavior and adverse outcomes.

Previously, ROS was detected at the apical surface of thyrocytes, indicating a relatively high level of this oxidizing agent in the thyroid gland^3,4^. More recently, the observation that somatic mutations are present in higher levels in the rat thyroid gland has further confirmed that the thyrocyte is under oxidative stress^5^. Unlike other oxidoreductases that generate ROS only as by-products along their specific catalytic pathways, NOXs family are ‘professional’ producers of ROS, as their primary function is to generate these molecules^6^. Among the NOXs family NOX4 is expressed at a high level in human thyroid tumours and is controlled at the transcriptional level by thyroid Stimulating Hormone(TSH) unlike dual oxidases(DUOXs)^7,8^. Heterodimerization of NOX4 with the p22phox is able to increase ROS production^9^. However, the source of ROS, possibly contributing to various disorders associated with enhanced proliferation in PTC, involved in NOX4 has only recently begun to be clarified.

The metabolism of malignant tumors can be explained with Warburg effect, a metabolic shift from oxidative phosphorylation (OXPHOS) to glycolysis in tumor cells^10^. Hypoxic microenviroment induces the shift and stabilizes hypoxia-inducible transcription factors(HIFs), which associated with the regulation of glycolysis and the shift to a suppression of oxidative metabolism^11^. However, its stabilization is required for the ROS production, which happen to depend directly on NOX4 expression in PTC.

In the present article, we describe the role of NOX4 play a part not only in PTC proliferation but also in cellular metabolism in hypoxic PTC. The aim of the study was to analyze the sources of mROS in hypoxia sustained by NOX4 and to explore the contribution of glycolysis induced by NOX4/p22phox on PTC proliferation and metabolism.

## Results

### TPC-1 proliferation is inhibited due to NOX4 knockdown

**Figure 1:**
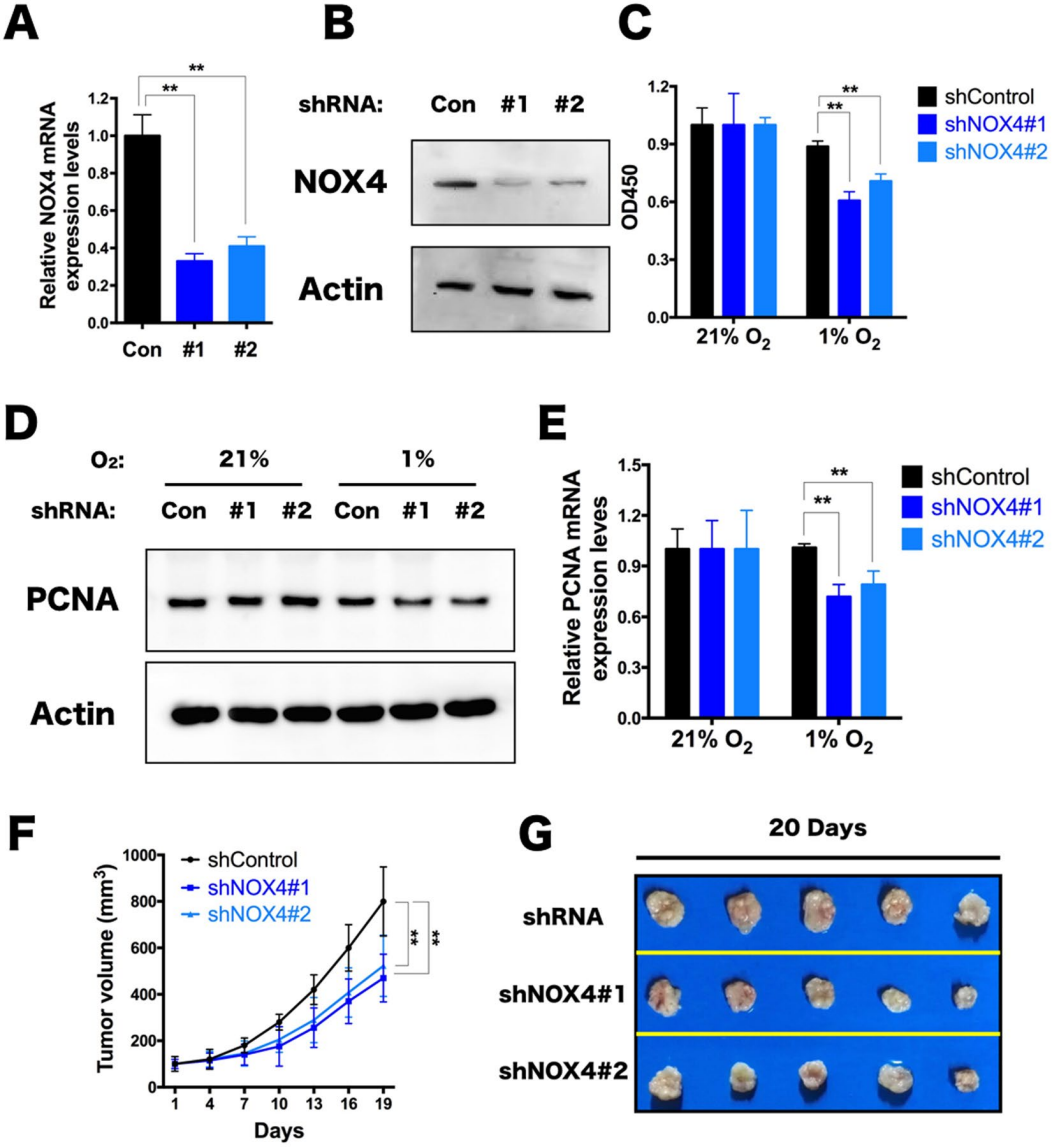
NOX4 Knockdown results in inhibition of TPC-1 Proliferation. (**A,B**) Transcriptional expression of NOX4 in TPC-1 cells after 48 hours treated with lentiviral transduction particles targeting NOX4 mRNA (**A**). Protein expression level of NOX4 after 72 hours treated with lentiviral transduction particles targeting NOX4 mRNA (**B**). Con for shNOX4 control lentivirus, #1 for shNOX4#1 lentivirus, and #2 for shNOX4#2 lentivirus. **P < 0.01. (**C)** Viability assay for TPC-1 cells expressing shControl or shRNA against NOX4 (shNOX4#1,#2) which were cultured in normoxia (21% O_2_) and hypoxia (1% O_2_) respectively for 48 hours using CCK8 assay (n = 8). **P < 0.01. (**D,E)** Western blot for normoxia (21% O_2_) and hypoxia (1% O_2_) in TPC-1 cell clones after infected with either shNOX4 control lentivirus and shNOX4#1and shNOX4#2 lentivirus (**D**). The blots were quantified using ImageJ software (n = 3). **P < 0.01. (**F,G)** TPC-1 cells transduced with shNOX4 control or two NOX4-directed shRNAs were injected subcutaneously in the flanks of nude mice. Tumor growth was quantified with a caliper at the indicated time intervals for 20 days (**F**). After the measurement, these mice were euthanized and then stripped of the subcutaneous transplantation tumor to take pictures at 20 days (**G**). Data were analyzed using the two-sided unpaired Student’s t test. Mean ± SEM. **p < 0.01.

To investigate the role of NOX4 in the proliferation of thyroid cancer cells, two NOX4-knockdown cell stains were established by short hairpin RNA(shRNA) and NOX4 was severely interfered in the strain TPC-1 (Fig. 1A,B). Then we found that the viability of the knockdown cells using cell counting kit-8(CCK8) did not have a obvious change under common conditions (Fig. 1C). Considering the growth microenvironment of tumor cells, cells was put in the hypoxic incubator (1% O_2_) to mimic growth condition. Compared to control cell strain in hypoxia, the growth of shRNA targeting cells was decreased by nearly 30% (Fig. 1C), and very similar phenotypes also appeared in other two papillary thyroid cancer cell lines K1 and BCPAP (Supplementary Fig. S1).

To further investigate the causes of cell proliferation decline under hypoxia, the protein immune blot after lysating cells showed that, the proliferating cell nuclear antigen (PCNA) expression level in the NOX4 knockdown cells under hypoxia was downregulated (Fig. 1D,E), highlighting the effect of NOX4 in regulating the growth of thyroid cancer cells under hypoxic microenvironment. Otherway, NOX4 knockdown cells exhibited little change when using both Casepase 3 detection and Annexin V & PI staining after hypoxic treatment (Supplementary Fig. S2), delineating the *in vitro* reduction in growth observed is not due to active cell death through apoptosis.

To determine *in vivo* tumorigenesis, cells were implanted into athymic nude mice. After about 1 weeks, the two NOX4 knockdown cell strains gave rise to smaller tumors, which suggests that loss of NOX4 provides a necessary genetic event resulting in tumorigenesis (Fig. 1F,G). These data suggest that NOX4 is essential for thyroid tumor maintenance.

### NOX4 is required for mROS level upon hypoxia

**Figure 2:**
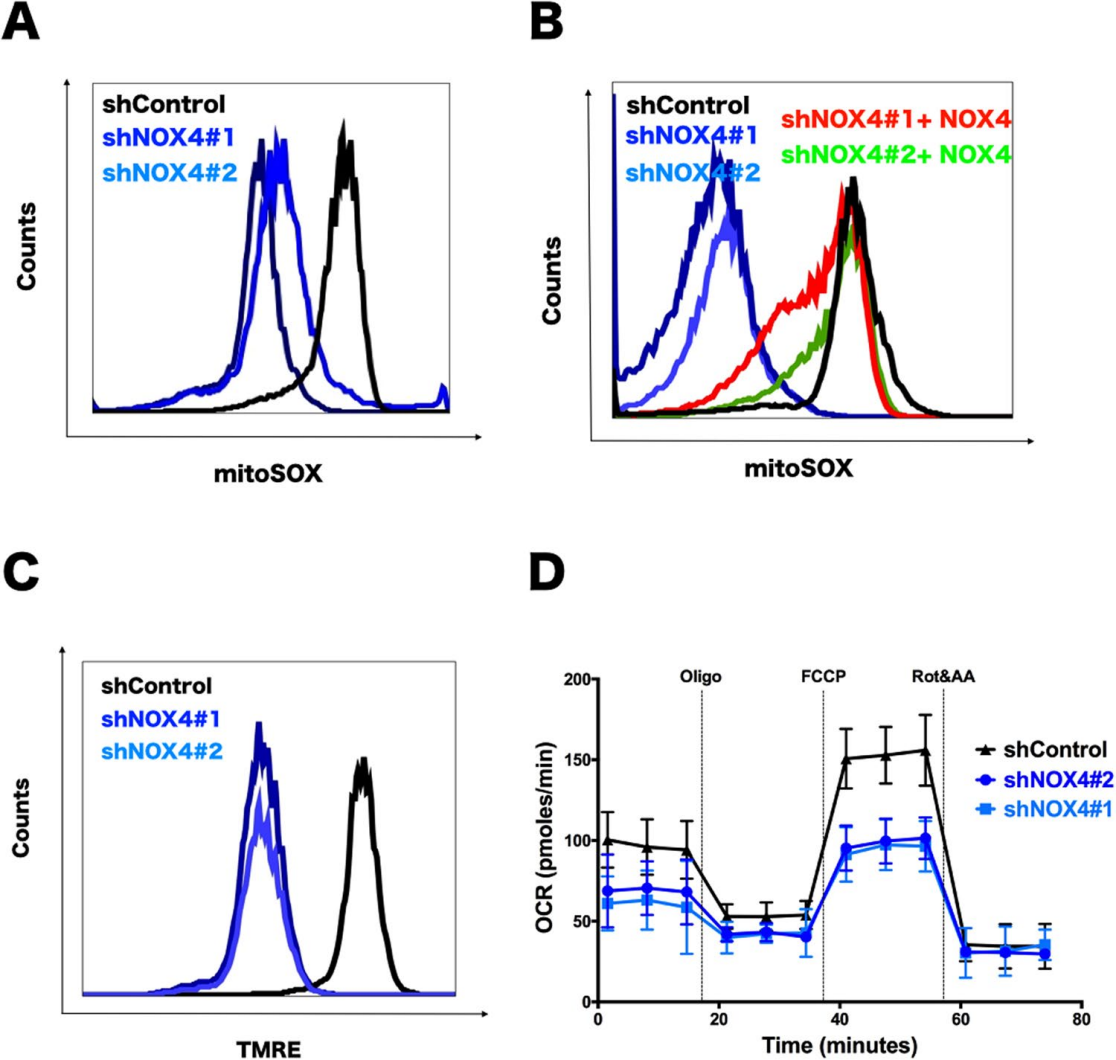
NOX4 is required for mitochondrial function in thyroid cancer during hypoxia. (**A,B)** Mitochondrial ROS level was measured with MitoSox staining by flow cytometry analysis in TPC-1 cells cultured in 1% O_2_ for 48 hours (**A**). NOX4 transfection can rescue mROS production decreasing after cell was treated with 1% O_2_ for 48 hours (**B**). (**C**) Mitochondrial membrane potential assessed by TMRE (50 nM) staining of NOX4 knockdown TPC-1 cells. (**D**) Whole-TPC-1 cell oxygen consumption rate (OCR) in different groups. The dotted lines indicate the time of adding oligomycin (1 μM), FCCP (1 μM) and rotenone and antimycin A (Rot/AA, 1 μM each).

NOX4 was described to play a role in superoxide and hydrogen peroxide production, so we evaluated its contribution of increased intracellular ROS using mitochondrial indicator, mitoSOX, upon hypoxia in thyroid cancer cells and investigate the reason why thyroid cancer proliferation was decreased by NOX4 inhibition upon hypoxia *in vitro*. Unsurprisingly, knockdown of NOX4 led to largely lowered mROS compared to the control levels in hypoxia (Fig. 2A). Importantly, when the vector of NOX4 overexpression was transfected into NOX4 knockdown cells, the level of mROS was recovered in hypoxia (Fig. 2B). This manipulation was consistent with the previous reports about relationship of NOX4 and ROS^12^.

However, if the reduction in proliferation in fact due to lowered ROS, the effect that treating NOX4 knockdown cells with low doses of hydrogen peroxide(H_2_O_2_) is able to rescue previous decreased proliferation *in vitro* should theoretically be observed. To verify this conjecture, H_2_O_2_ or an H_2_O_2_ generating enzyme, glucose oxidase, with a concentration gradient was added to the NOX4 knockdown TPC-1 cells during hypoxia. As a result, we found that either H_2_O_2_ or glucose oxidase with relative lower concentration can stimulate cell proliferation, but high concentration of both inhibits cell growth (Supplementary Fig. S3). The possible reason is that as a double-edged sword, H_2_O_2_ with exorbitant concentration is easy to cause oxidative damage and then counteract the effect of its stimulation.

As NOX4 is located in mitochondrial, we asked if NOX4 would affect the mitochondrial activity by staining the cells with a probe, TMRE, to monitor the membrane potential of mitochondrial. Using flow cytometry, the results showed that, the mitochondrial membrane potential was decreased when NOX4 was deleted in hypoxia, suggesting potent relevance between mitochondrial membrane potential and mROS under hypoxia in thyroid cancer (Fig. 2C). Consistent with previous reports, mitochondrial metabolism, indicated by oxygen consumption rate (OCR), was down-regulated in the basal and maximum respiration when NOX4 was knocked down relative to the control cells pretreated with hypoxia (Fig. 2D). Altogether, NOX4 reduces mitochondrial activity by decreasing oxygen consumption, mROS and mitochondrial membrane potential under hypoxia in thyroid cancer.

### NOX4 maintains HIF1α stability in thyroid tumor cells

HIF1, the heterodimer of HIF1a and HIF1b, is the primary driver of increased glycolysis and lactate production during hypoxia. Under conditions of low oxygen, HIF1α is stabilized by mROS and promotes transcription of many genes crucial for the cellular response to hypoxia^13,14^. Consequently, cells lacking HIF1α fail to upregulate glycolytic enzymes and lactic acid production in response to hypoxia. Given the mROS regulation by NOX4 under hypoxia in thyroid cells, in addition that the way of HIF1α stabilization is dependent on mROS, we reasoned that the mechanism by which NOX4 regulates HIF1α involves mROS in thyroid cells.

**Figure 3:**
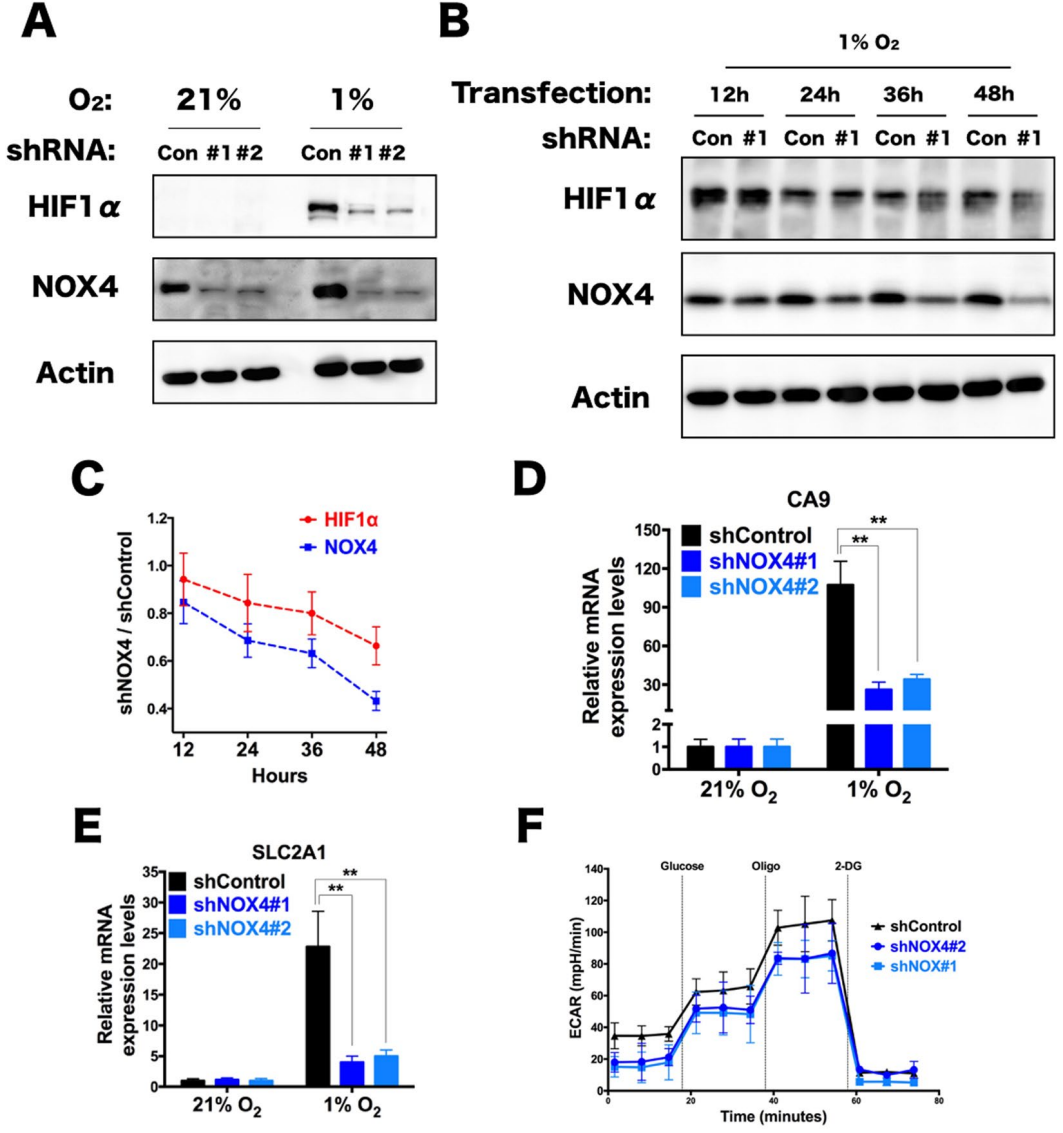
HIF1α stabilization and glycolysis require NOX4 expression. (**A**) Immunoblots of NOX4 knockdown cells and its Control respectively cultured under 21% O_2_ and 1% O_2_ for 48 hours. (**B,C**) Immunoblot of TPC-1 cells grown in 1% O2 for 48 hours after tranfected with shRNA vector targeting NOX4 for the indicated time points (**B**). Levels of HIF1α and NOX4 quantified using Image J and normalized to actin (**C**). (**D,E**) TPC-1 cells expressing shControl or shRNA against NOX4 (shNOX4#1,#2) were cultured in 21% O_2_ and 1% O_2_ (48 hours), and the fold change in CA9 (**D**) and SLC2A1 (**E**) levels was measured by qRT-PCR. (**F**) The extracellular acidification rate (ECAR) was measured in TPC-1 cells expressing shControl or shRNA against NOX4 (shNOX4#1,#2) cells treated with 1% O_2_ (48 hours). Data show a representative experiment (from 3 independent experiments). Error bars ± SD (n = 3).

To test this idea we first investigated whether NOX4 directly modulates HIF1α stability under hypoxic conditions. Generally in the presence of high oxygen, HIF1α was rapidly degraded and difficult to measure in cells, but HIF1α was detectable under hypoxia conditions. Surprisingly, NOX4-deficient cells during hypoxia demonstrated low levels of HIF1α relative to control cells (Fig. 3A). Notably, HIF1α was decreased at each of the time points in cells after transfected with NOX4 knockdown vector under hypoxia, suggesting that the reason for destabilization of HIF1α was due to gradually reduced NOX4 expression (Fig. 3B,C). Furthermore, the HIF1α targets genes, carbonic anhydrase 9(CA9) and solute carrier family 2 member 1(SLC2A1), were significantly decreased in NOX4 knockdown cells compared to control cells during hypoxia (Fig. 3D,E).

Tumor cells exhibits metabolism characterized by high glycolysis especially when lacking of oxygen, HIF1α triggers tumor cells to upregulate glycolytic enzymes and lactic acid production in response to hypoxia^15^. Consistent with HIF1α destabilization by NOX4 knockdown under hypoxia, bioenergetics profiling of NOX4-deficient TPC-1 revealed glycolytic loss less than the control cells: Extracellular acidification rate (ECAR) decreased obviously, suggesting NOX4 plays an essential role in the maintenance of glycolysis during TPC-1 hypoxic metabolic process (Fig. 3F). Based these results, it is suggested that NOX4 is critical for glycolytic metabolism via HIF1α signaling under hypoxia in thyroid caner cells.

Finally, to test whether a stabilized version of HIF1α can rescue NOX4 knockdown phenotypes in hypoxia regarding reduced ROS and reduced proliferation, a recombined HIF1α expression vector was used from ADDGENE which used pcDNA3 as a vector backbone^16^. Strikingly, we found that HIF1α rescued the decreased proliferation of NOX4 knockdown cells, but not restoring the mROS levels (Supplementary Fig. S4), reflecting that NOX4 regulates thyroid cancer cell growth dependent on mROS-HIF axis, rather than HIF-mROS.

### p22phox is also requires for TPC-1 proliferation via mROS-HIF pathway

**Figure 4:**
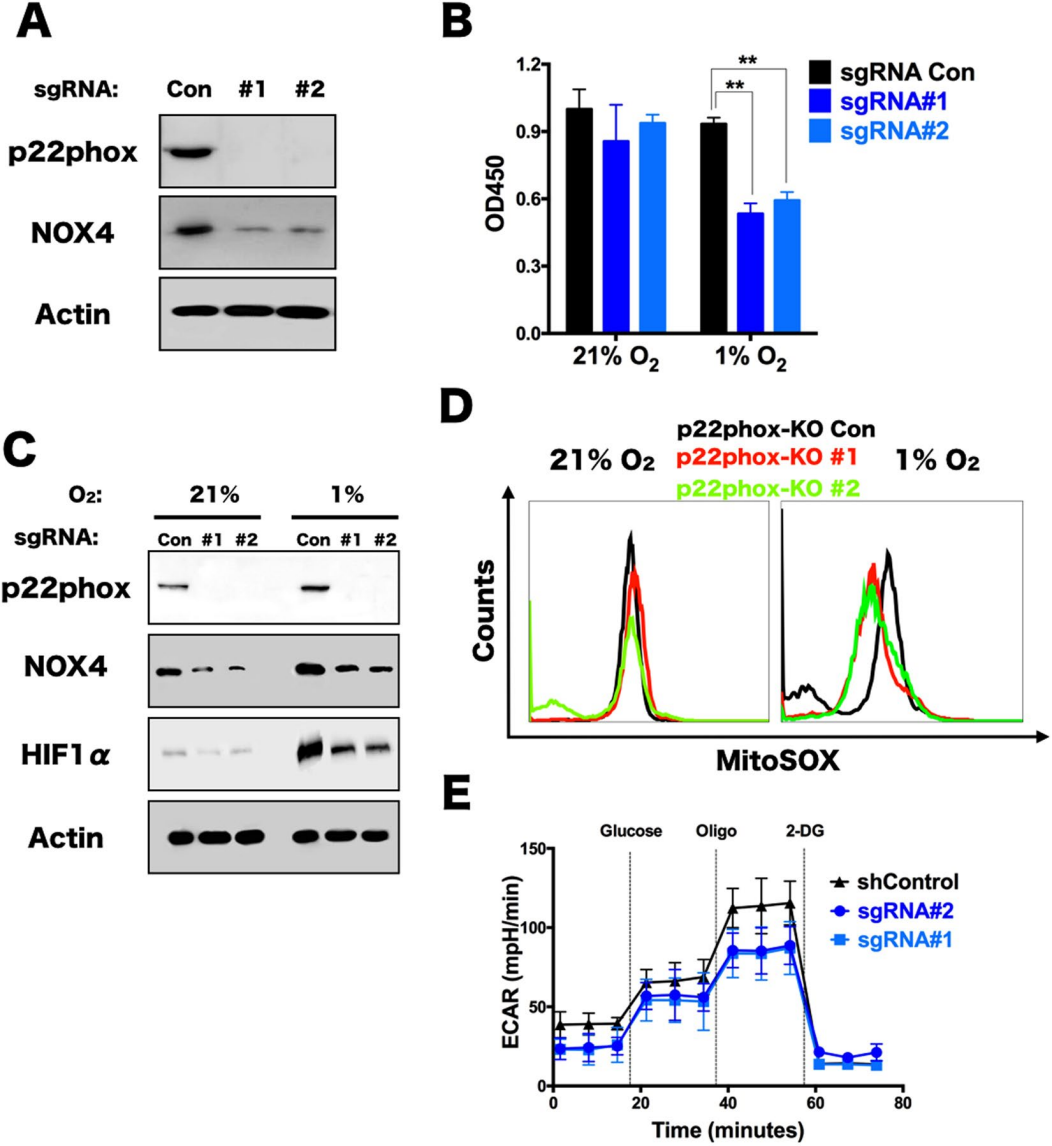
p22phox is essential for NOX4 expression and TPC-1 proliferation. (**A**) Immunoblots of p22phox knockout cells and its Control in TPC-1. (**B**) Viability assay for TPC-1 cells expressing sgRNA control(LentiCRIPSR V2.0) or sgRNA against p22phox (#1, #2) which were cultured in normoxia (21% O_2_) and hypoxia (1% O_2_) respectively for 48 hours using CCK8 assay (n = 8). **P < 0.01. (**C**) Immunoblots of p22phox knockout cells and its Control respectively cultured under 21% O_2_ and 1% O_2_ for 48 hours. (**D**) Mitochondrial ROS level was measured with MitoSox staining by flow cytometry analysis in TPC-1 cells expressing sgRNA control (LentiCRIPSR V2.0) or sgRNA against p22phox (#1, #2) cultured in 1% O_2_ for 48 hours. (**E**) ECAR was measured in TPC-1 cells expressing expressing sgRNA control (LentiCRIPSR V2.0) or sgRNA against p22phox (#1, #2) cells treated with 1% O_2_ (48 hours). Data show a representative experiment (from 3 independent experiments). Error bars ± SD (n = 3).

NADPH oxidases are differentially activated by a whole host of binding partners, including p22phox, p40phox, p47phox/NOXO1, p67phox/NOXA1 and Rac^17^. The NOX4 enzyme has been shown to be activated solely by the p22phox binding partner. It was demonstrated that NOX4 and p22phox expression are upregulated in thyroid cancers, linking NOX4-dependent ROS generation to cancer development or progression^12^. Therefore, in order to study the NOX4 activator, p22phox as with NOX4, can assist in the level of mROS in thyroid cancer cells upon hypoxia, p22phox knockout in TPC-1 cells was prepared mediated by CRISPR/Cas9 system. As shown in the immunoblot (Fig. 4A), NOX4 was positively regulated by endogenously p22phox expression, suggesting a role of p22phox in NOX4 associated signaling in thyroid cancer.

Further, we wanted to know the functional effect of p22phox on the proliferation of thyroid cancer cells in hypoxia. Cell Counting kit-8(CCK8) assays displayed that TPC-1 proliferation with p22phox knockout in hypoxia was slowed down rather than normoxia (Fig. 4B), suggesting it is functionally consistent with NOX4 in regulating the proliferation of hypoxic thyroid cancer cells. Another issue that concerned us was the regulatory relationship between P22phox and HIF1α, therefore, immunoblot was performed that HIF1α was significantly decreased in p22phox knockout TPC-1 cells compared to the control cells in hypoxia (Fig. 4C). Consistently, the mROS level and glycolytic flux were both reduced in hypoxia once upon p22phox deletion (Fig. 4D,E). These data illustrate that p22phox regulates its functional heterodimer, NOX4, further sustain glycolytic flux via mROS-HIF1α pathway in hypoxic condition.

Moreover, to understand whether add-back of NOX4 can rescue the effects of loss of p22phox, beacause they heterodimerize and this is important as p22 inhibition also results in decreased NOX4 abundance. However, the results shown that NOX4 overexpression had little effect on the mROS production, glycolytic rate and deceased cell proliferation in p22phox null cells during hypoxia (Supplementary Fig. S5), directly showing to us that NOX4 regulates proliferation of papillary thyroid carcinoma cells through a way dependent on p22phox-NOX4 heterodimer complex.

## Discussion

NOX family proteins are highly regulated enzymes that in recent years have been implicated in an extremely wide array of physiological and pathophysiological processes. More and more studies have shown that NOX is closely related to the development of cancer, part of them reported that inhibition of NOX activity can inhibit the growth of tumor and promote the death of cancer cells^12,18–21^. And so forth, increased NOX4/p22phox in cancer might be related to a higher proliferation rate and tumor progression. In this study we demonstrate that NOX4 regulates thyroid cancer cell proliferation both in TPC-1 cells and xenografts. Interestingly, the latter showed contradictory results, that is, the lower proliferation markers and the level of apoptosis. Previously, Nox4 was regarded as a target in the treatment of clear cell RCC because it is essential for full HIF2α expression and activity in 786–0 renal tumor cells, even in the absence of functional VHL^18,22^. Our study shows that NOX4 additionally controls HIF1α stabilization by the mROS production. The decreased mROS in NOX4 null cells contributes to increased HIF1α destabilization. Significantly, loss of NOX4 in human papillary thyroid cancer cells decreases HIF1α targeting genes such as SLC2A1 and CA9, highlighting the potential importance of NOX4-mediated metabolic reprogramming in thyroid tumor. This idea is further validated by the finding that p22phox represses the glycolysis via NOX4-mROS-HIF1α axis in TPC-1 cells. Taken together, we provide a mechanism whereby NOX4 or p22phox functions as a tumor glycolytic regulator which is required for thyroid carcinoma proliferation.

ROS is no longer viewed as solely damaging or signaling agents. Indeed, cancers cells take advantage of ROS producted from normal cell processes in order to drive tumor growth. Thus, heightened levels of ROS, especially mROS, are used to induce pro-tumorigenic signaling^23^. In our study, we demonstrated that NOX4 and its functional parter, p22phox, are specific sourse of the mROS in hypoxic microenvironment. However, we found that rescue of p22phox knockout cells with NOX4 CDS expression can not restore mROS production, which reflected that at least NOX4, must form a heterodimer with p22phox in regulating ROS production, ECAR and proliferation in PTC. Further studies should determine how much the contribution rate of NOX and p22phox protein respectively to mROS and total ROS in thyroid cancer cells exactively are, and how the sensitivity of cell response to thyroid related hormones is when NOX4 and p22phox protein levels are altered by someway.

Hypoxia is a major feature of tumor cells *in vivo*, as there is often an imbalance between their rapid proliferation rate and the growth of new blood vessels that supply O_2_^24^. In fact, in order to support growth beyond about one millimeter in diameter, tumors must recruit the expansion of new vessels to ensure proper nourishment^25^. As explained previously, cells stabilize HIF under hypoxia in an mROS-dependent manner. Since mROS is known to be both necessary and sufficient for HIF stabilization under hypoxia, we report that NOX4 and p22phox in papillary thyroid carcinoma are necessary for mROS production during hypoxia. On this basis, the other mitochondrial function is consequently altered, such as mitochondrial membrane potential and oxygen consumption, both of which represents the basic ability level of mitochondrial. The findings suggest that NOX4 has the function of regulating the basic state of mitochondria, which may be one of the reasons why the proliferation of thyroid cancer cells slows down.

At the current state of data, we provide the first evidence that NOX4 functions as a glycolytic regulator coupling the metabolism to the papillary thyroid carcinoma proliferation. In the future, we further observe the effect of the NOX4 as a molecular target for thyroid cancer from the perspective of metabolism.

## Materials and Methods

### Cells and Cell Counting Kit-8

The human papillary thyroid carcinoma cell lines TPC-1 cell line(ATCC) was cultured in RPMI 1640 medium(Thermo fisher, 11875168) supplemented with 10% fetal bovine serum(Thermo fisher, 10437-028) and antibiotics in a 37 °C incubator with 5% CO_2_. The papillary thyroid cancer cell line BCPAP and K1 was obtained from Jiehui Technology (YunNan Province), and maintained in DMEM containing 10% fetal bovine serum, 100 U ml^−1^ penicillin and 100 U ml^−1^ streptomycin under a humid atmosphere of 5% (v/v) CO_2_ at 37 °C.

Cell viability was determined by Cell Counting-Kit 8 assay (MCE). The assay was performed in 96-well plates and the absorbance was measured at 450 nm in a microplate reader (Thermo Varioskan Flash) after 4 hours of incubation. The assays were performed with N = 8 and repeated 3 times.

### Xenografts

For xenografts, 5 × 10^6^ TPC-1 cells of shNOX4 and shControl stable single-cell knockdown clones were injected subcutaneously into nude mice (6 to 9 weeks-old males, Charles river). There were 5 mice in each group of which was injected with shControl cells and two shNOX4 cells. Tumor size was measured every 3 days, and tumors were dissected and weighed after 21 days. The average tumor volume was calculated as Volume = L × W^2^ × 0.52.

Tumors were fixed in 4% paraformaldehyde and embedded in paraffin. Sections were stained with hematoxylin and eosin (H&E) in accordance with standard procedures. Immunohistochemistry was performed using tunel staining(Beyotime, C1091) and Ki67 antibody (abcam, ab833) according to manufacturer instructions.

### Generation of shNOX4 stable single-cell knockdown clones

Lentiviral transduction particles for vector control or human shNOX4 were used to generate stable knockdown clones in TPC-1 cells. Lentiviral transduction particles (Sigma Aldrich product#: TRCN0000046089 and TRCN0000046090) was used with the infected enhancer polybrene(Sigma). TPC-1 cells were infected with Lentiviral shNOX4 or shControl as per the manufacturer’s instructions. 48 hours post infection, 5000 cells were selected and passed into 96-well plates in puromycin (2000 ng/ml) antibiotic for selection and maintained in puromycin (1000 ng/ml).

### CRISPR/Cas9 mediated p22phox knockout clones

CRISPR/Cas9 system was used to disrupt the endogenous p22phox in TPC-1 cells. The annealed double-stranded single-guide RNAs (sgRNAs) downstream of the p22phox start codon were cloned into LentiCRISPRv2 (Addgene) by BsmBI(NEB). Two sgRNAs targeting human p22phox (sgRNA#1 5′-AACGAACAGGCGCTGGCGTC-3′ and sgRNA#2 5′-GGCCATGTGGGCCAACGAAC-3′) were effective to produce mutation of respective gene in genomic DNA. The reconstituted vector transfected into 293T cells using lipofectamine 3000 (Invitrogen) along with pMD2.G and psPAX2 packaging vectors to produce p22phox KO control(sgRNA Control), p22phox KO#1 (sgRNA #1)and p22phox KO#2(sgRNA #2) lentivirus. TPC-1 cells were infected with these Lentiviral and 48 hours post infection, 5000 cells were selected and passed into 96-well plates in puromycin (2000 ng/ml) antibiotic for selection and maintained in puromycin (1000 ng/ml).

### Western blotting

Cells were harvested and lysed with RIPA (20 mM Tris PH7.5, 150 mM NaCl, 1% Triton X-100, 2.5 mM sodium pyrophosphate, 1 mM EDTA, 1% Na3VO4, 0.5 μg/ml leupeptin, 1 mM PMSF). For immunoblotting, proteins from whole-cell lysates were resolved by 10 or 12% sodium dodecyl sulfate-polyacrylamide gel electrophoresis (SDS-PAGE) and then transferred to PVDF membranes. Anti-NOX4(Novus), anti-p22phox(Santa Cruz), anti-HIF1α(BD science), anti-Actin(Beyotime), anti-PCNA(CST) and anti-caspase 3(abcam) antibodies were used at 1:1000 and secondary antibodies (Beyotime) conjugated with horseradish peroxidase (HRP) were used at 1:1000 dilutions in 5% non-fat dry milk. After the final washing, PVDF membranes were exposed for an enhanced chemiluminescence assay using the Gel imaging instrument (BIO-RAD ChemiDoc MP).

### qPCR

Total RNA was extracted using the TRIZOL (Invitrogen) following manufacturer’s instructions and then reversed transcribed using reverse transcriptase (Takara). Quantitive PCR was performed on the BIO-RAD CFX 96 system using SYBR Green Master mix (Takara). Reactions were performed with 125 ng of template cDNA. Transcript levels of genes were normalized to a reference index of housekeeping genes (ACTB). Primers for ACTB were used as an internal control. The primers used were as follow. NOX4: 5′-GAAAA CCCGG CTCTG GGTAG-3′ (forward), 5′-TGATC CTCGG AGGTA AGCCA-3′ (reverse); PCNA: 5′-GGATA CCTTGG CGCTA GTATT T-3′ (forward), 5′-CACAG CTGTA CTCCT GTTCT G-3′ (reverse); CA9: 5′-TCAGC CGCTA CTTCC AATAT G-3′ (forward), 5′-TCAGC ATCAC TGTCT GGTTA AA-3′ (reverse); VEGFA: 5′-CAGGA CATTG CTGTG CTTTG-3′ (forward), 5′-CTCAG AAGCA GGTGA GAGTA AG-3′ (reverse); ACTB: 5′-GGACC TGACT GACTA CCTCA T-3′ (forward), 5′-CGTAG CACAG CTTCT CCTTA AT-3′ (reverse).

### Measurement of Mitochondrial ROS and potential

mROS production in trypsinized TPC-1 cells was measured using the mitoSOX(Molecular Probes, 36008) and TMRE cell fluorescent dye (50 nM) as supplier’s instructions. Briefly, Apply 1.0 mL of 5 μM MitoSOX to cover TPC-1 cells, and incubate cells for 10 minutes at 37 °C, protected from light. Wash cells gently three times with PBS and detected by Flow Cytometry at excitation/emission maxima of 510/580 nm. Mitochondrial membrane potential was evaluated by fluorescence of the potential dependent TMRE probe(Thermo fisher, T668). Briefly, tripsinized cells were incubated with 50 nM TMRE for 15 min in the dark, after which the media was replaced and the fluorescence was measure by flow cytometry. Cells were collected and suspended in PBS and analyzed by flow cytometer (BD FACSCalibur) and the data were analyzed using FlowJo 10.0.7 software.

### OCR and ECAR Measurement

Oxygen consumption rate(OCR) and extracellular acidification rate (ECAR) were measured using a Seahorse XF24 analyzer (Seahorse Bioscience). Briefly, Oxygen consumption rate (OCR) and extracellular acidification rate (ECAR) were determined using the MitoStress kit (Seahorse Biosciences) and Glycolysis Stress kit (Seahorse Biosciences) according to the manufacturer’s standard protocol. OCR and ECAR were calculated and recorded by the Seahorse XF24 software. All assays were performed with N = 3 or more per condition and repeated 3 times.

### Statistical analysis

Student’s t-test was used in all cellular experiments and the results from three independent experiments are presented as mean SEM. In addition, all the mRNA expression data represent the mean values with standard error of the mean (SEM). The differences in mRNA expression data were compared using the ratio t-test. All statistical analyses were performed using the GraphPad Prism software. Asterisks denote statistical significance as follows: NS, P > 0.05; *P ≤ 0.05; **P ≤ 0.01.

### Statement

We confirm that all methods were carried out in accordance with relevant guidelines and regulations of the Municipal Committee for health and family planning of Mianyang, China. We confirm that all experimental protocols in this study were approved by the Third Hospital of Mianyang (Sichuan mental health center), China.

## Electronic supplementary material

Supplementary Information
