# Supplementary material for: NADPH oxidase NOX4 is a glycolytic regulator through mROS-HIF1α axis in thyroid carcinomas
Source: Sci Rep. 2018 Oct 26;8:15897. doi: 10.1038/s41598-018-34154-8 (PMC6203707; doi:10.1038/s41598-018-34154-8)
Supplement: Supplementary file 1 — Supplementary Information [file 41598_2018_34154_MOESM1_ESM.docx]

**Supplementary Figures**

**NADPH oxidase NOX4 is a glycolytic regulator through mROS-HIF1α axis in thyroid carcinomas**

Tang Ping^1,†^, Dang Hao^2,†^, Huang Jie^3^, Xu Tao^1^, Yuan Ping^1^, Hu Jun^1^, Sheng Jianfeng^1,*^

1. Otorhinolaryngology Head and Neck Surgery, The Third Hospital of Mianyang(Sichuan mental health center), No.190 The East Jiannan Road, Mianyang, 621000, Sichuan, People's Republic of China.
2. Department of Clinical Laboratory, The Third Hospital of Mianyang (Sichuan mental health center), No.190 The East Jiannan Road, Mianyang, 621000, Sichuan, People's Republic of China.
3. Jiehui Technology, the KunMing economic and Technological Development Zone, No.9 The Daxi Road, 650215，KunMing, Yunnan, People's Republic of China.

† These authors contributed equally to this work.

* Correspondence: ShengJianfeng0901@163.com

**Supplementary Figure1**

**B**

**A**


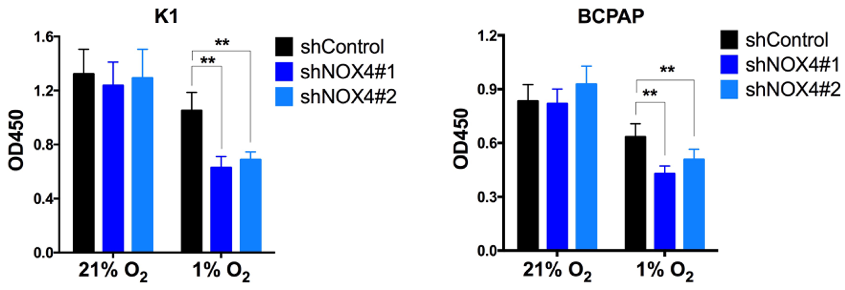


**Fig.S1.** Viability assay for K1 cells (A) and BCPAP cells (B) expressing shControl or shRNA against NOX4 (shNOX4#1,#2) which were cultured in normoxia (21% O_2_) and hypoxia (1% O_2_) respectively for 48 hours using CCK8 assay (n=8). **: P < 0.01.

**Supplementary Figure 2**

**A**


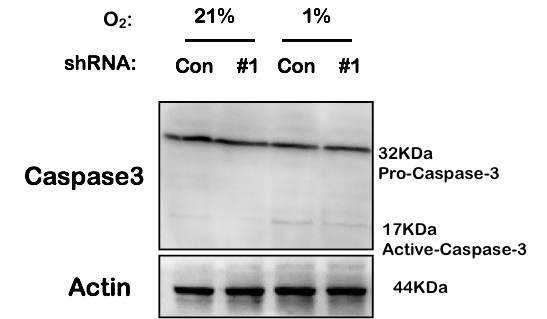


**B**

**
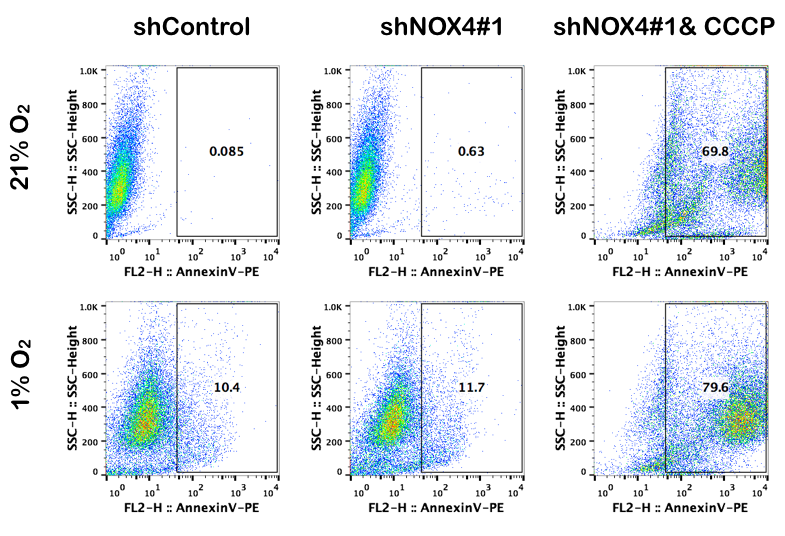
**

**Fig.S2. (A)** Western blot for normoxia (21% O_2_) and hypoxia (1% O_2_) in TPC-1 cell clones after infected with either shNOX4 control lentivirus and shNOX4#1and shNOX4#2 lentivirus. **(B)** Apoptosis in shNOX4 control cells and shNOX4 cells both in normoxia(21% O_2_) and hypoxia(1% O_2_) for 24 hours. As a positive control, cells were also treated with the apoptosis inducer CCCP(Carbonyl cyanide m-chlorophenyl

**Supplementary Figure 3**

**B**

**A**


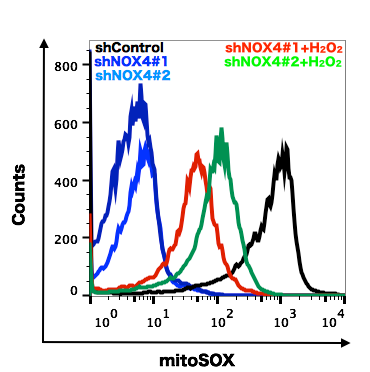

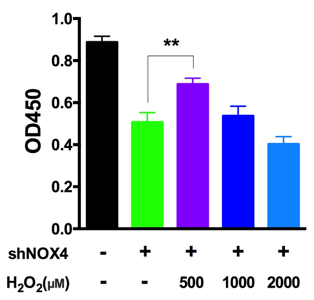


**D**

**C**


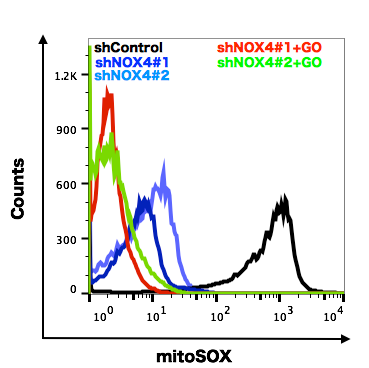

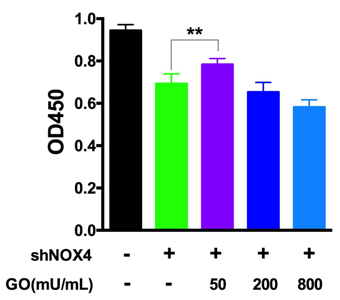


**Fig.S3. (A)** Mitochondrial ROS level was measured with MitoSox staining by flow cytometry analysis in TPC-1 cells cultured in 1% O_2_ for 48 hours with or without 500μM H_2_O_2_. **(B)** Viability assay for TPC-1 cells expressing shControl or shRNA against NOX4 (shNOX4#1) which were cultured in hypoxia (1% O_2_) respectively adding H_2_O_2_ with gradient concentration using CCK8 assay (n=8). **: P < 0.01. **(C)** mROS level was measured with MitoSox staining by flow cytometry analysis in TPC-1 cells cultured in 1% O_2_ for 48 hours with or without 50mU/mL glucose oxidase. **(D)** Viability assay for TPC-1 cells expressing shControl or shRNA against NOX4 (shNOX4#1) which were cultured in hypoxia (1% O_2_) respectively adding glucose oxidase with gradient concentration using CCK8 assay (n=8). **: P < 0.01.**Supplementary Figure 4**


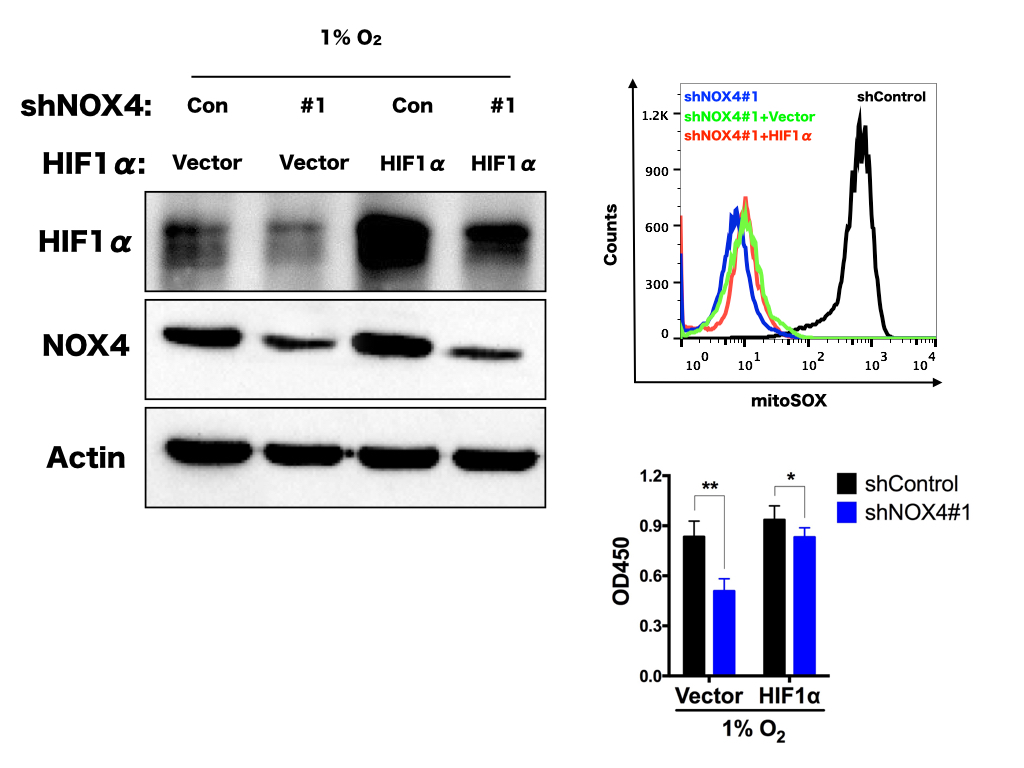
 **
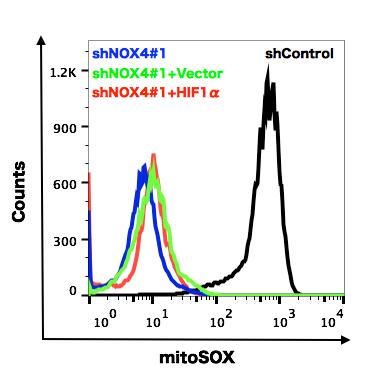
**

**C**

**B**

**A**


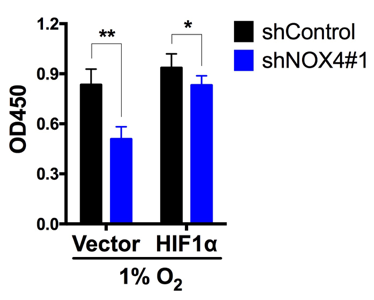


**Fig.S4. (A)** Western blot for normoxia (21% O2) and hypoxia (1% O2) in shControl and shNOX4 TPC-1 cells clones after transfected with either blank vector and HIF1α expression vector. **(B)**Mitochondrial ROS level was measured with MitoSox staining by flow cytometry analysis in TPC-1 cells cultured in 1% O2 for 48 hours with HIF1α expression vector and its control vector. **(C)** Viability assay for TPC-1 cells expressing shControl or shRNA against NOX4 (shNOX4#1) which were cultured in hypoxia (1% O2) respectively transfected with either blank vector and HIF1α expression vector using CCK8 assay (n=8). **: P < 0.01.

**Supplementary Figure 5**

**A**


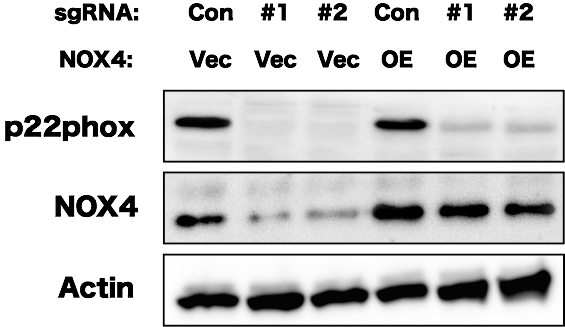


**B**

**
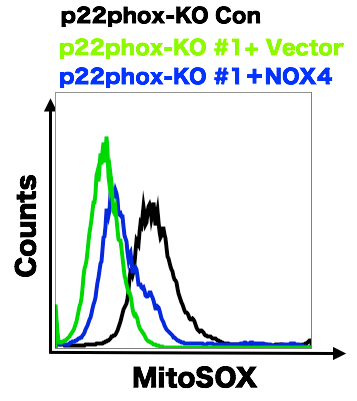

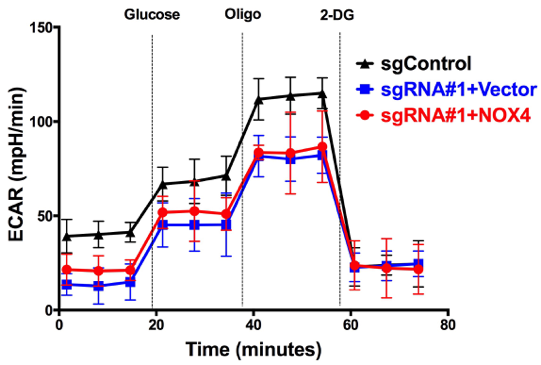
**

**C**

**
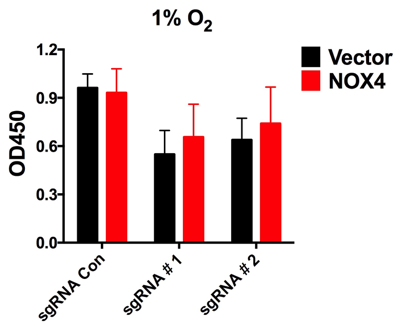
**

**D**

**Fig.S5. (A)** Immunoblots of p22phox knockout(sgRNA#1 and sgRNA#2) and its control TPC-1 cells respectively transfected with NOX4 recombined expressed plasmid and its blank vector under hypoxia. **(B)** Mitochondrial ROS level was measured with MitoSox staining by flow cytometry analysis in three indicated TPC-1 cells in hypoxia for 48hours. **(C)** ECAR was measured in three indicated cells treated with 1% O_2_ (48 hours). Data show a representative experiment (from 3 independent experiments). Error bars ± SD (n=3). **(D)** Viability assay for 6 groups of indicated TPC-1 cells cultured in hypoxia (1% O_2_) using CCK8 assay (n=8). **: P < 0.01.

**Supplementary Figure 6**

(The unprocessed original scans for all of the blots in your main figures and supplementary information above)

**
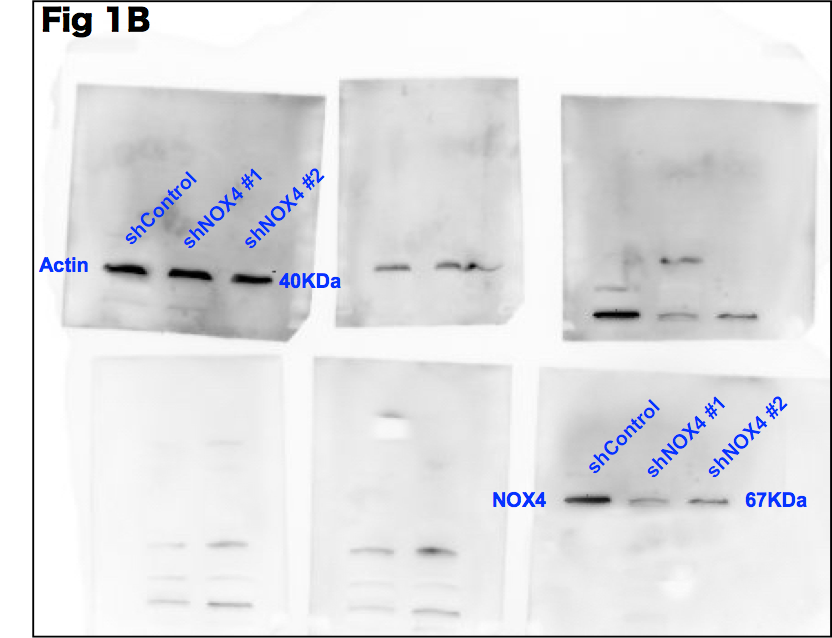
**

**
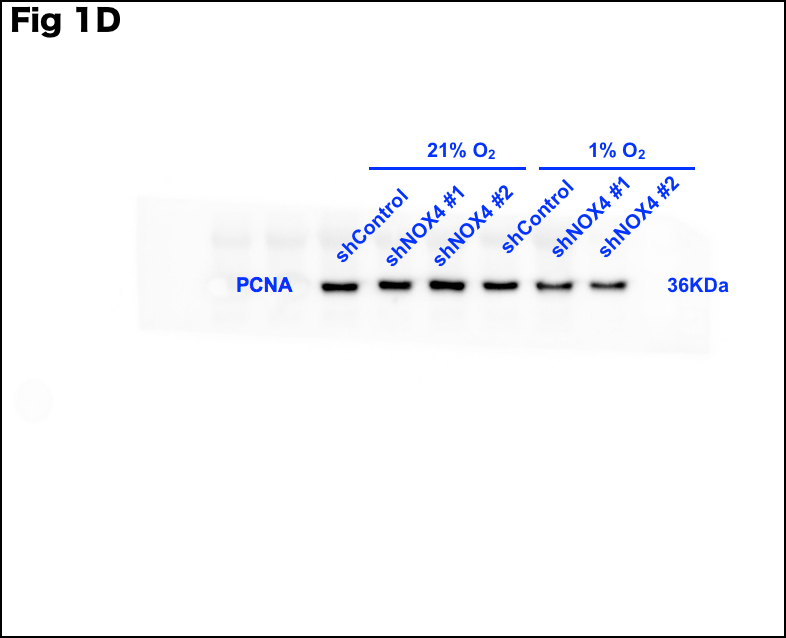
**

**
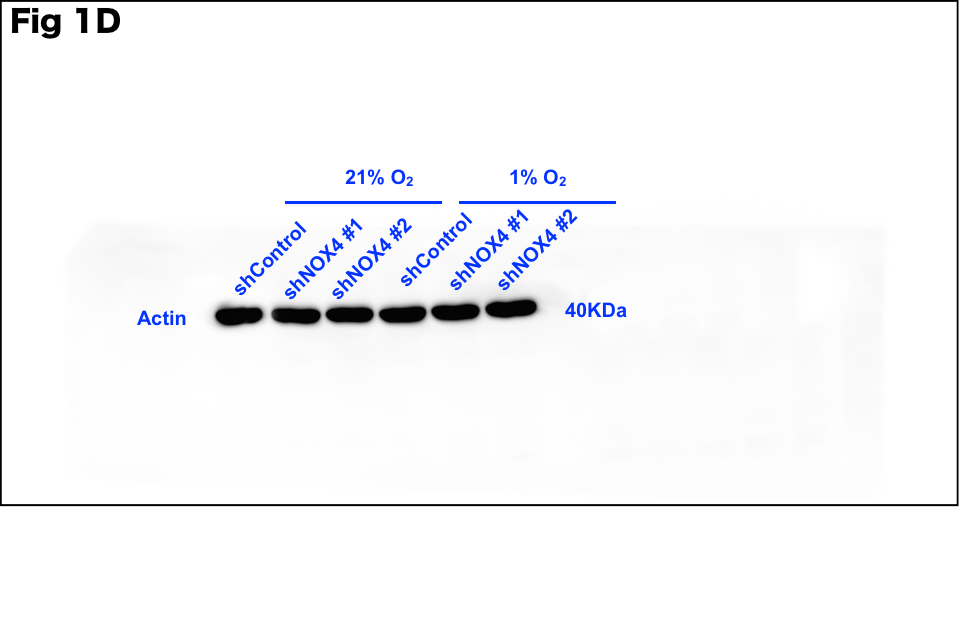
**

**
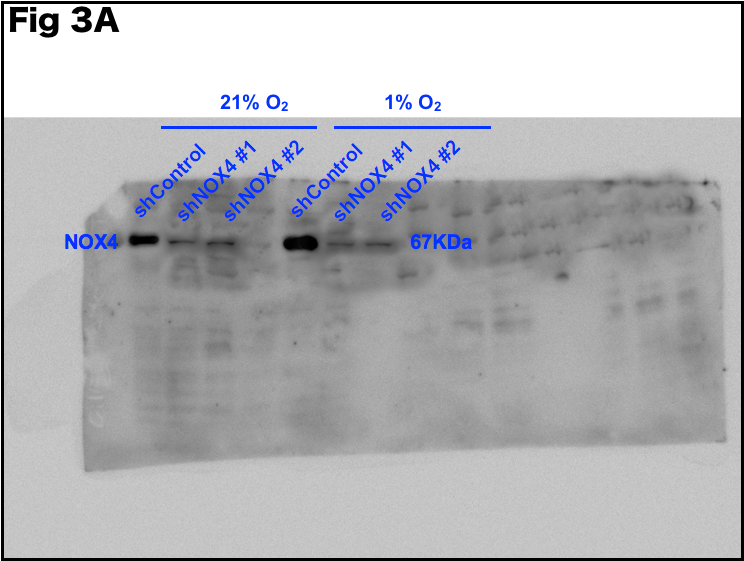
**

**
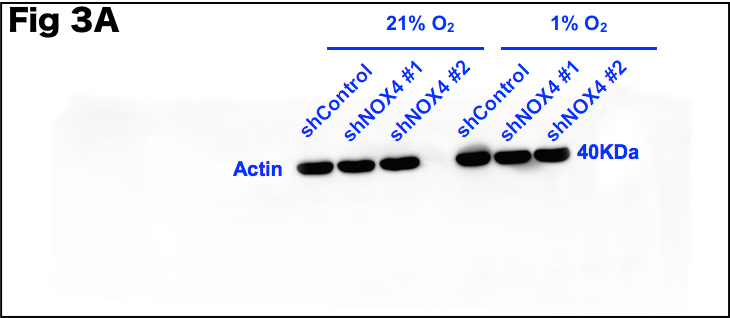
**

**
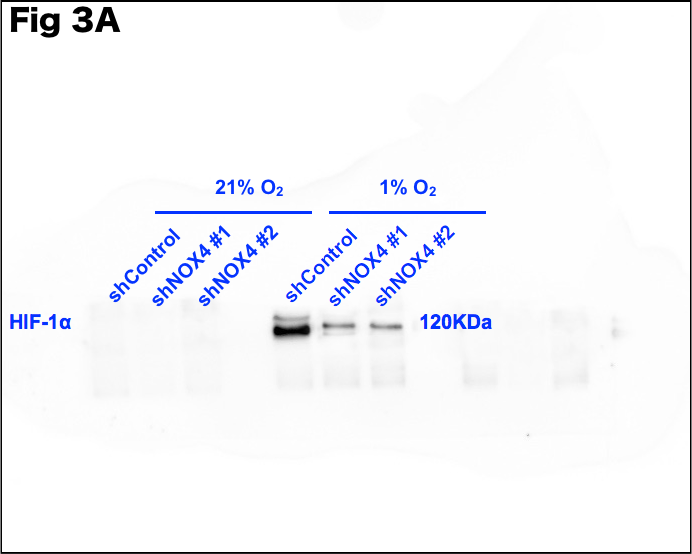
**

**
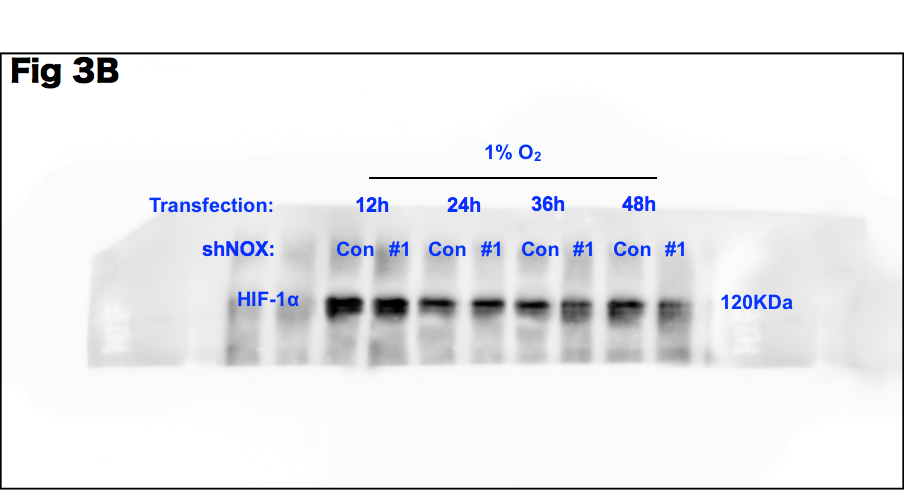
**

**
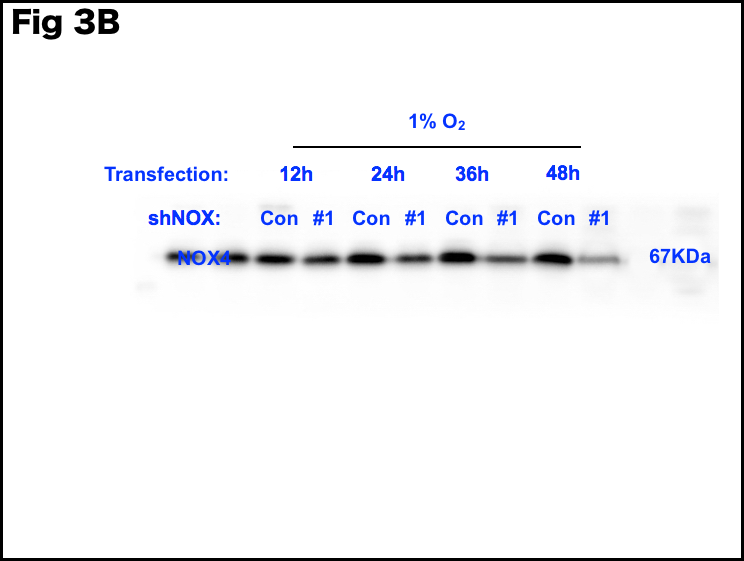
**

**
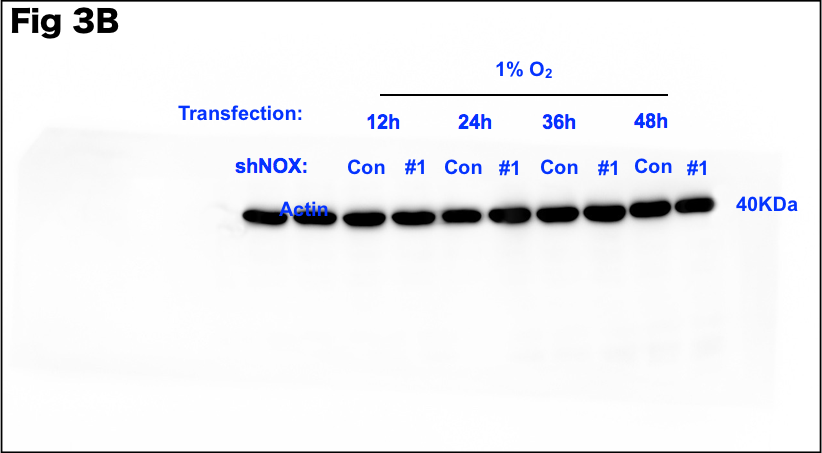
**

**
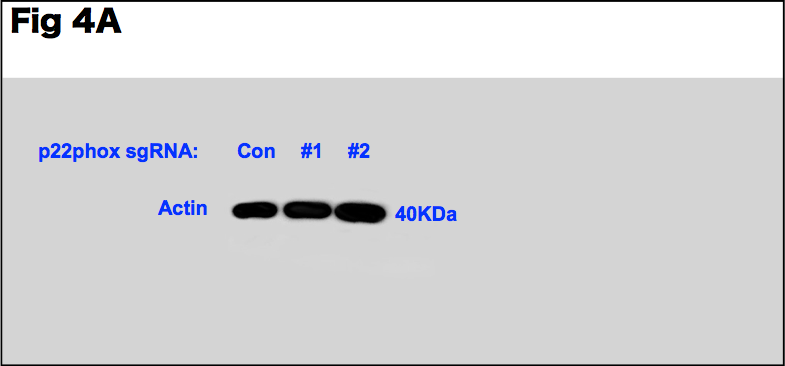
**

**
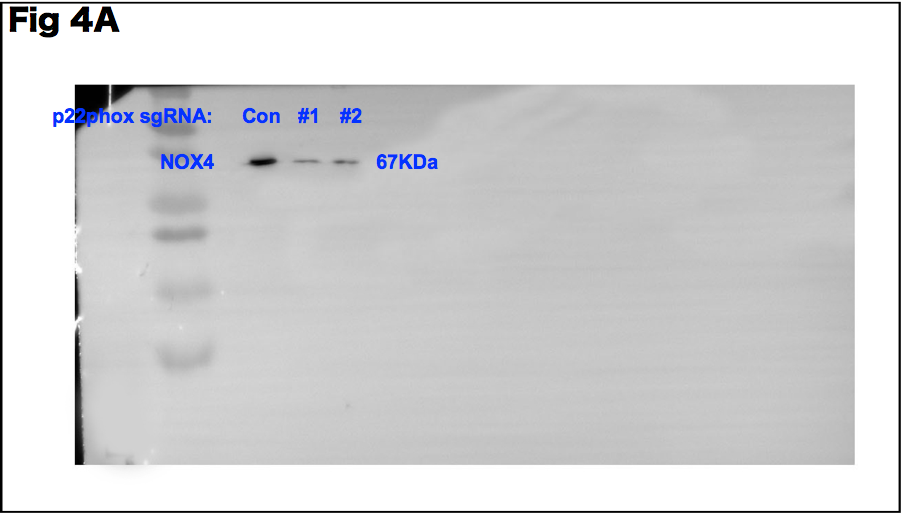
**

**
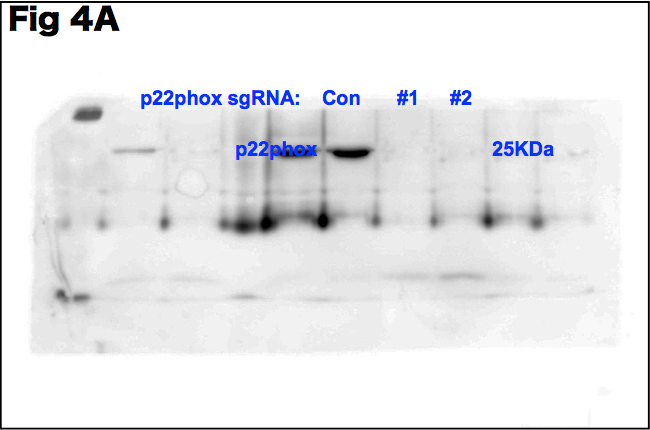
**

**
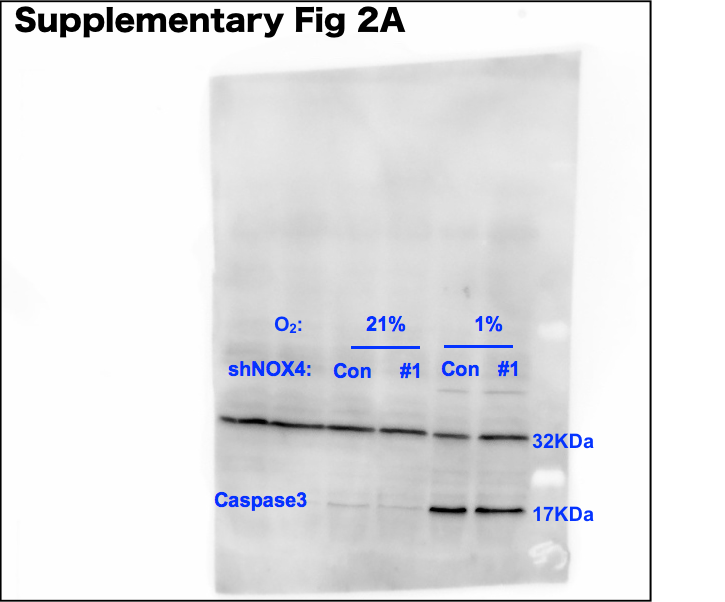
**

**
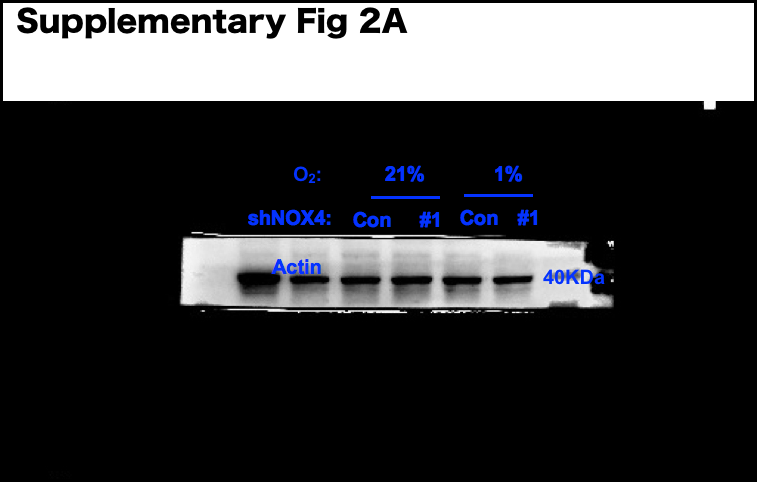
**

**
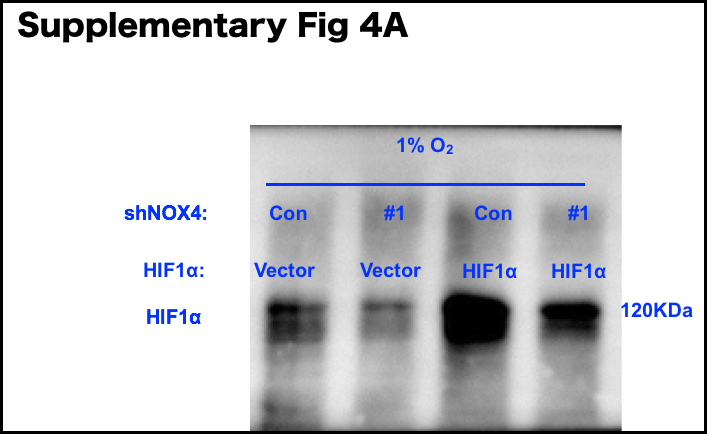
**

**
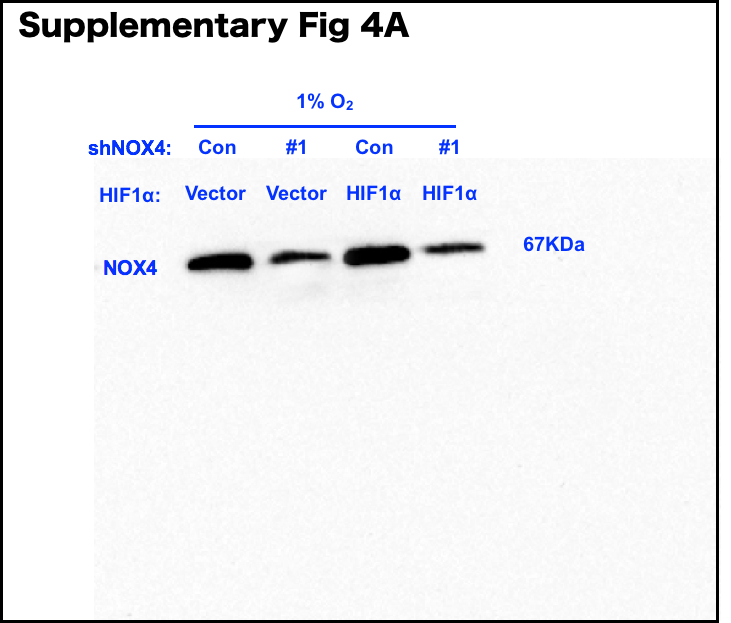
**

**
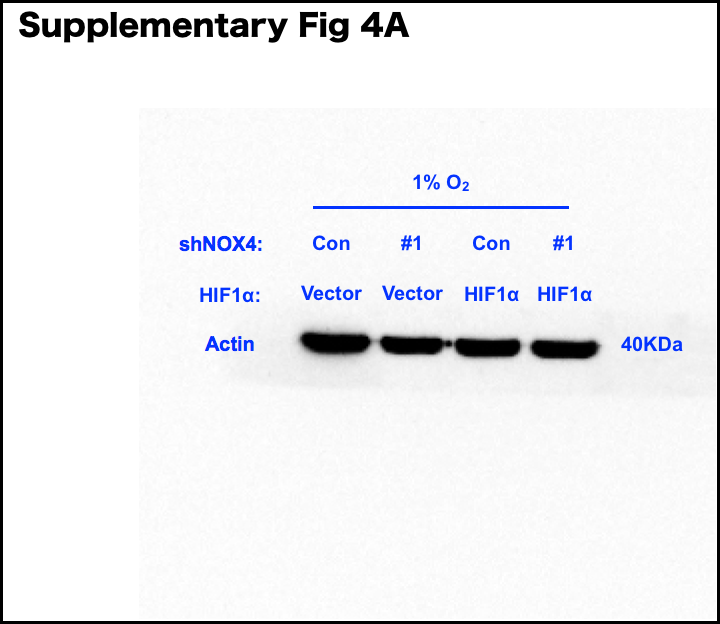
**

**
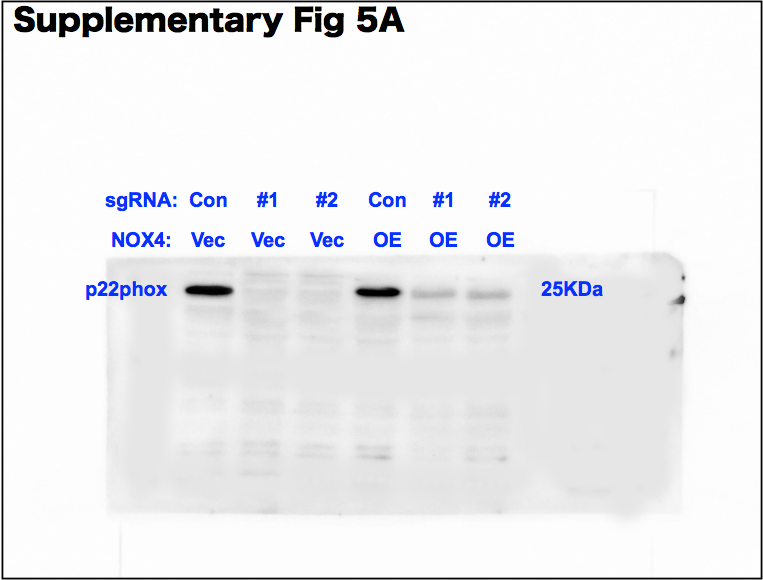
**

**
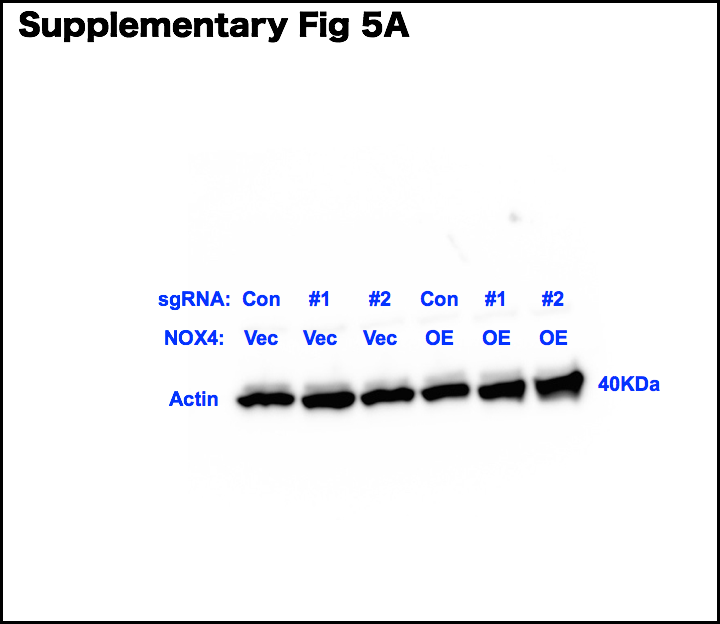
**

**
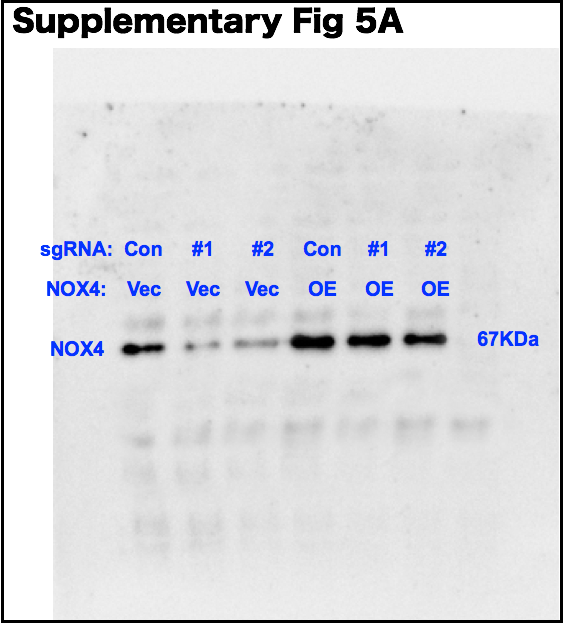
**
